# Supplementary figures and images for: De Novo Assembly and Annotation of 11 Diverse Shrub Willow (Salix) Genomes Reveals Novel Gene Organization in Sex-Linked Regions
Source: Int J Mol Sci. 2023 Feb 2;24(3):2904. doi: 10.3390/ijms24032904 (PMC9917877; doi:10.3390/ijms24032904)

**A**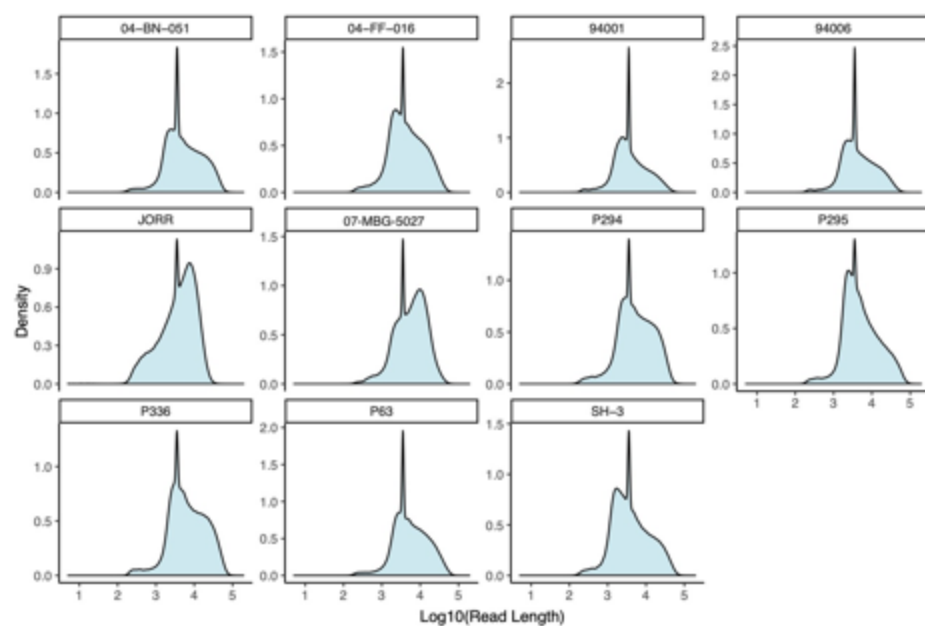**B**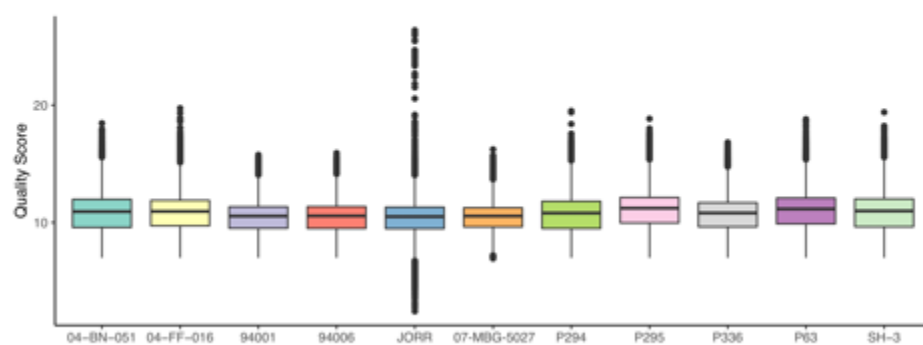

Supplement: Supplementary file 1 [file ijms-24-02904-s001.zip › Supplementary Figure S1.pdf]

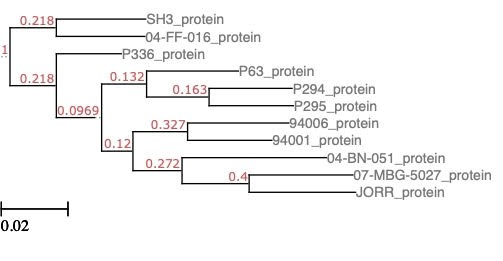

Supplement: Supplementary file 1 [file ijms-24-02904-s001.zip › Supplementary Figure S2.jpg]

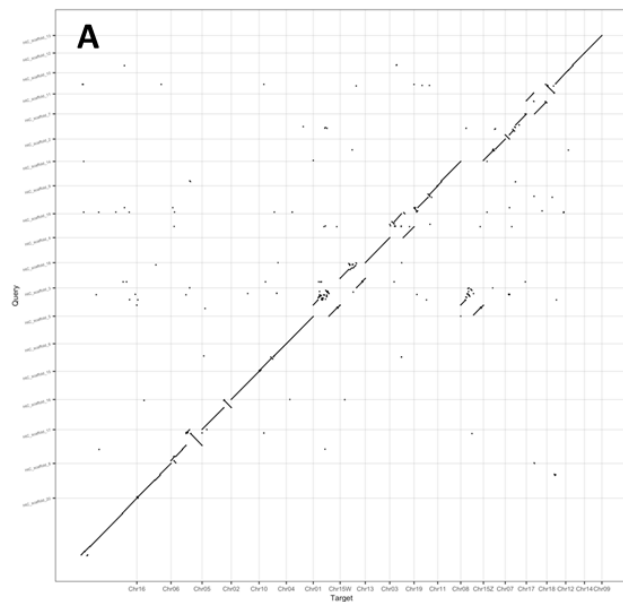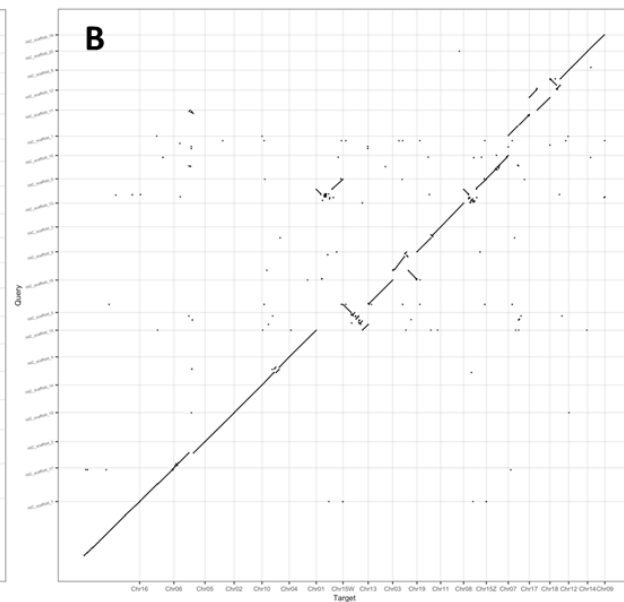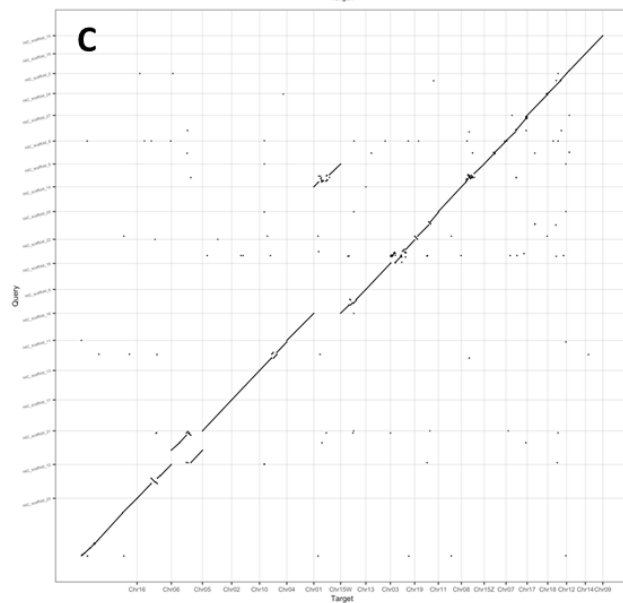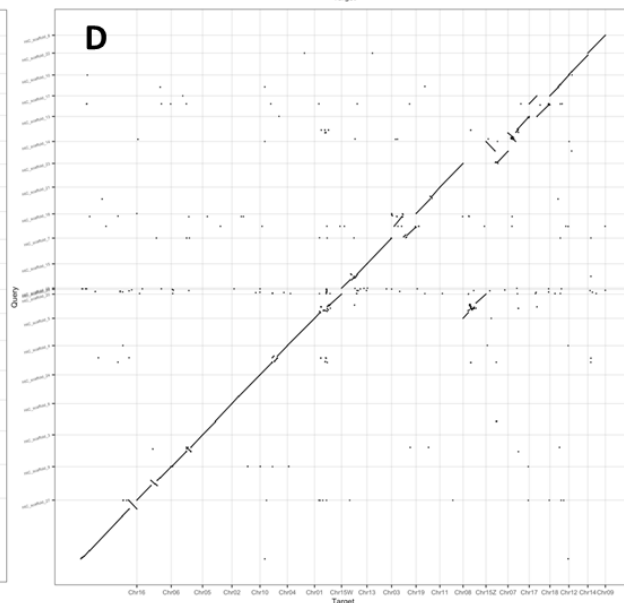



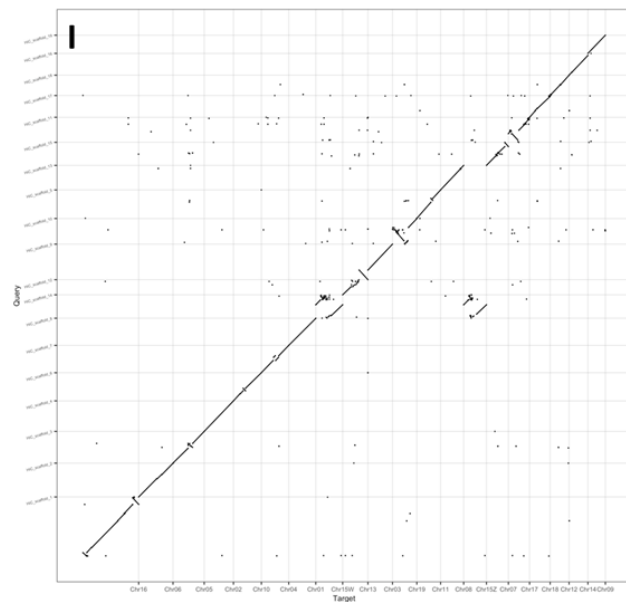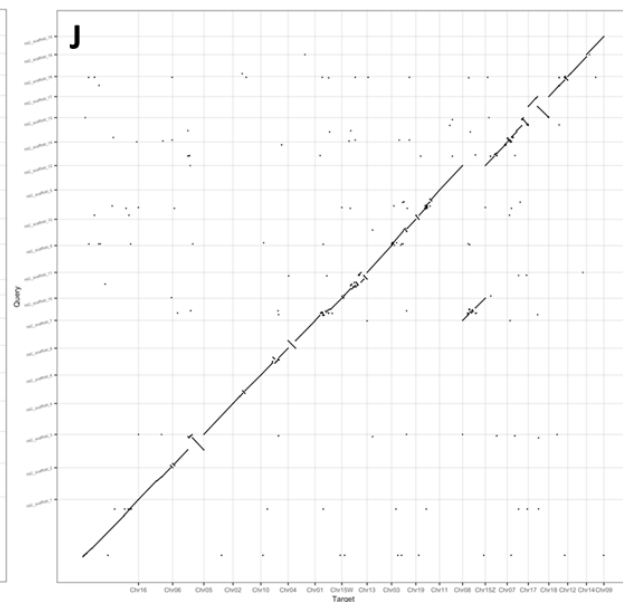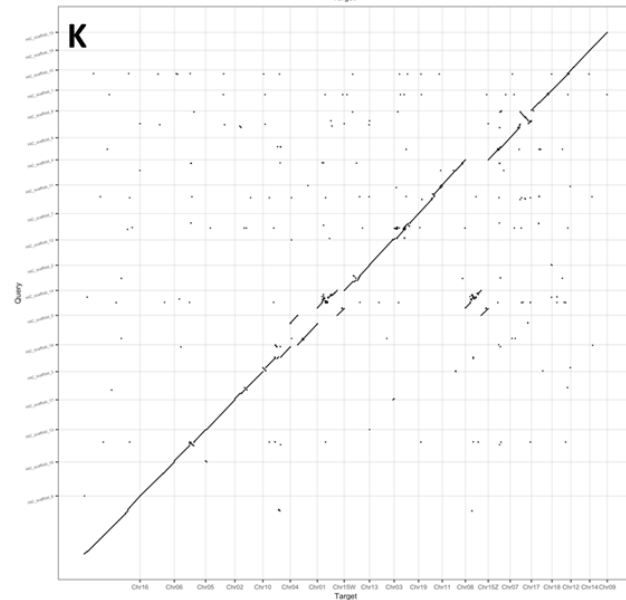

Supplement: Supplementary file 1 [file ijms-24-02904-s001.zip › Supplementary Figure S3.pdf]

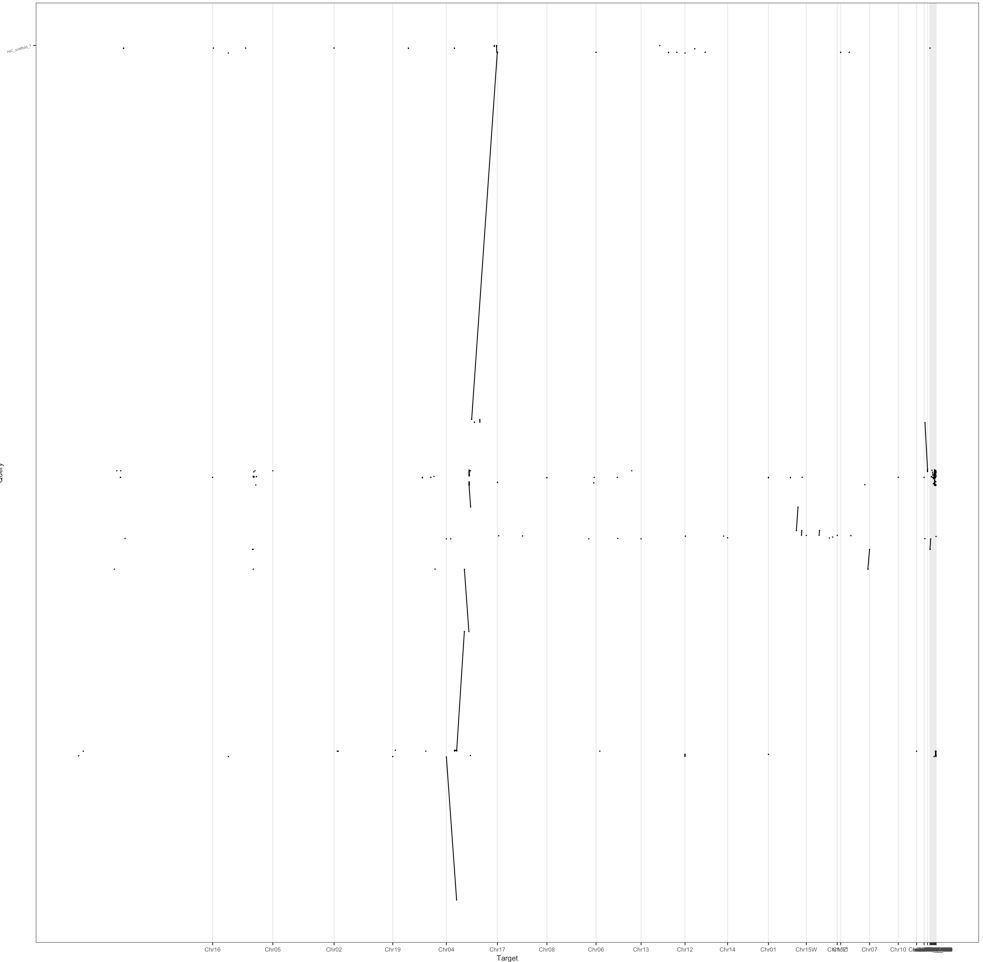

Supplement: Supplementary file 1 [file ijms-24-02904-s001.zip › Supplementary Figure S4.JPG]

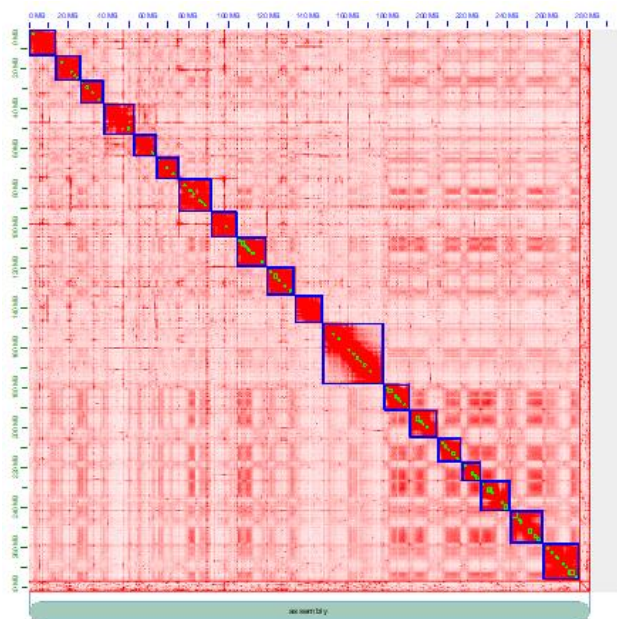

JORR

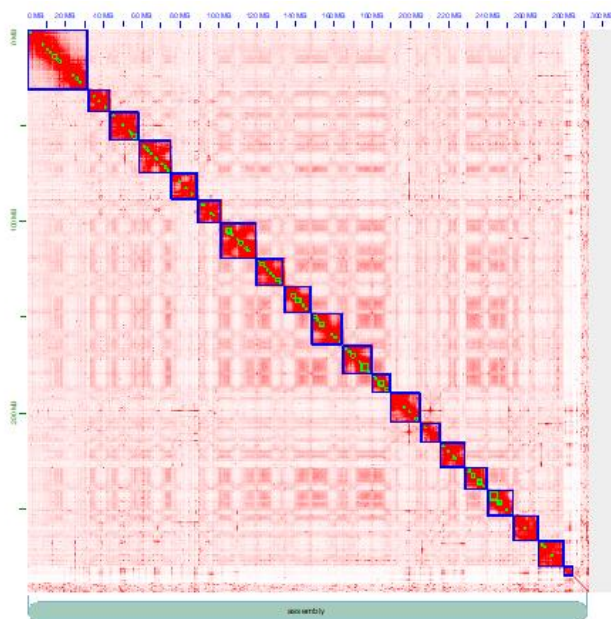

MBG

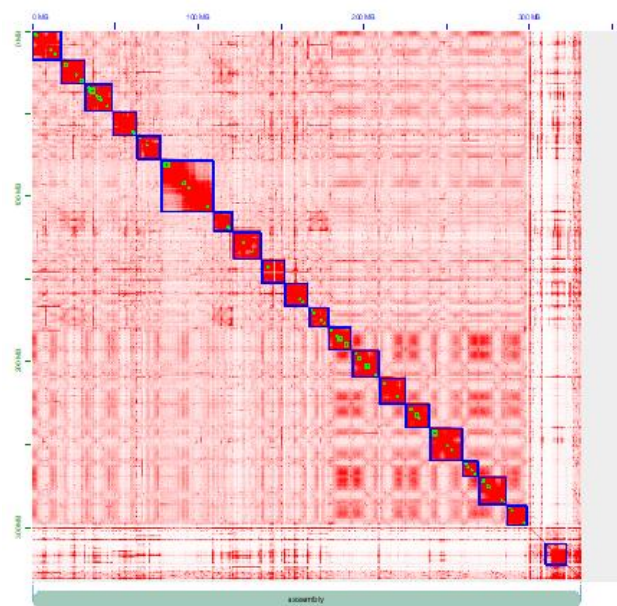

94001

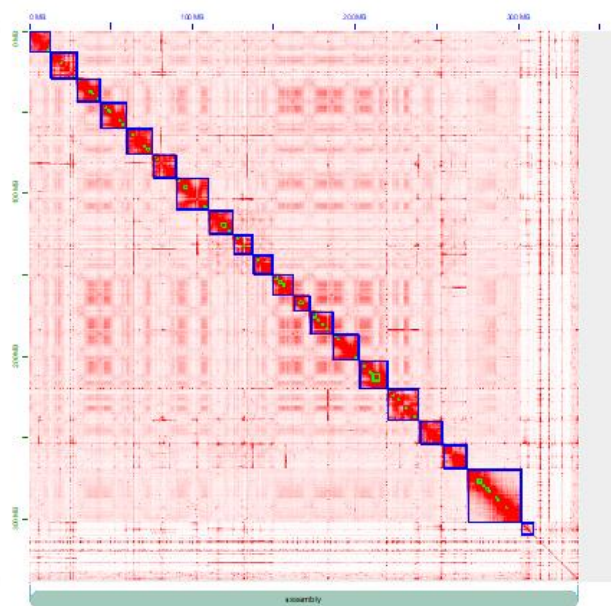

94006

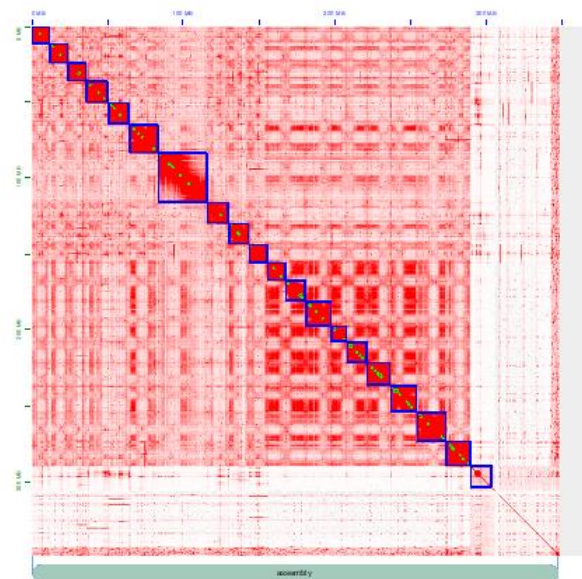

04-FF-016

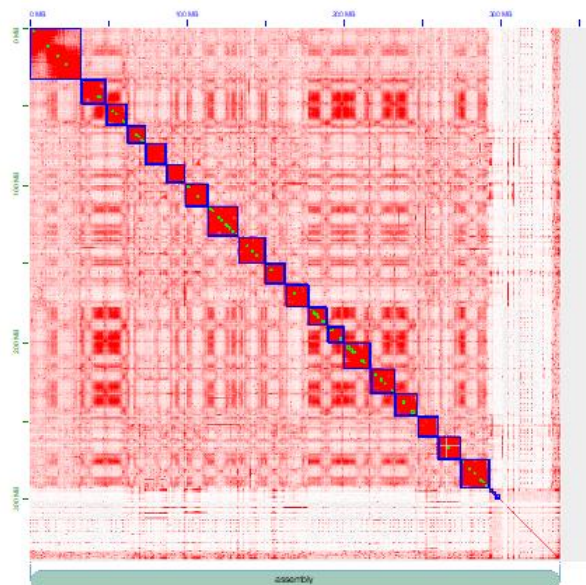

SH-3

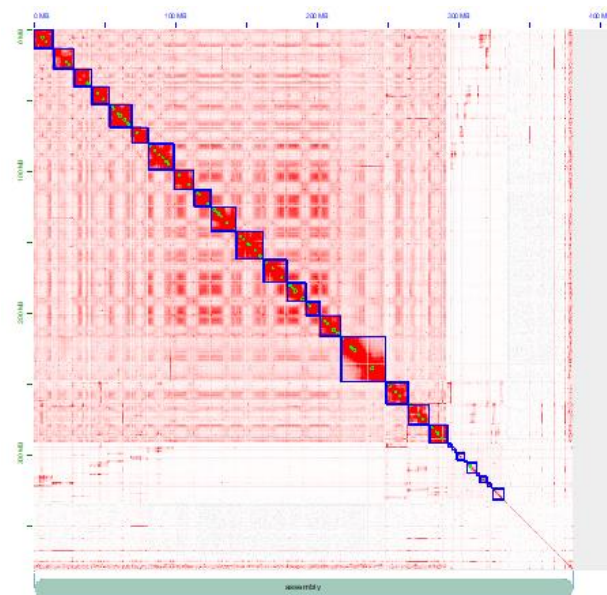

P295

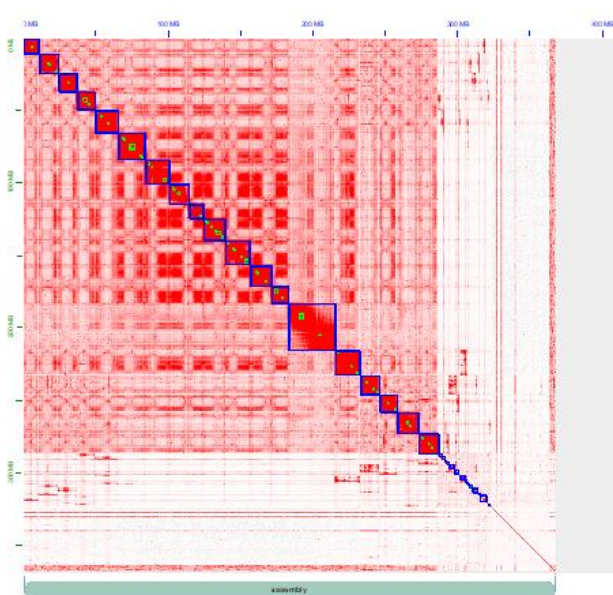

P63

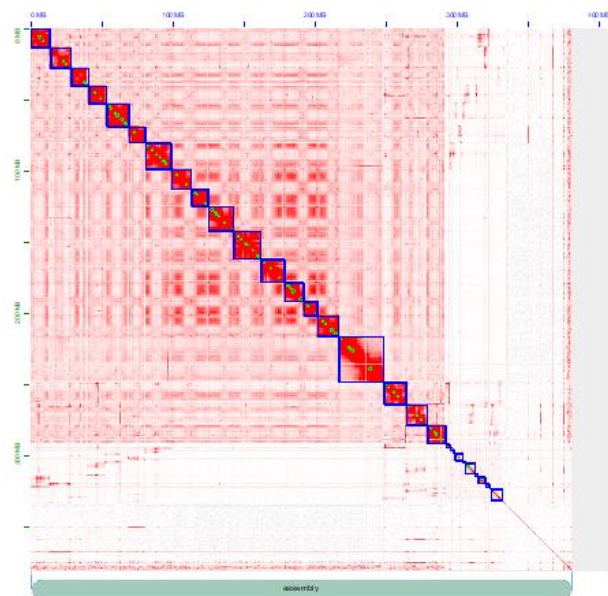

P295

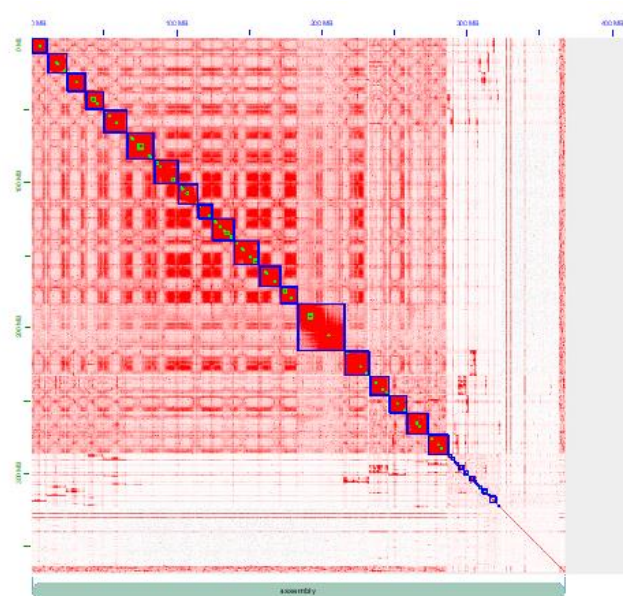

P63

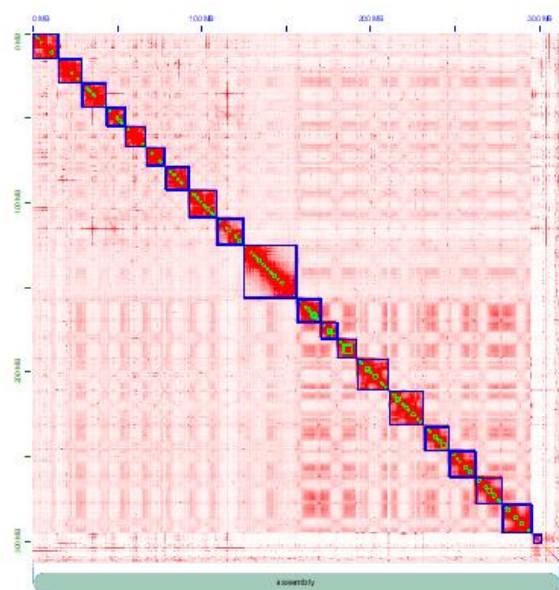

P336

Supplement: Supplementary file 1 [file ijms-24-02904-s001.zip › Supplementary Figure S5.pdf]

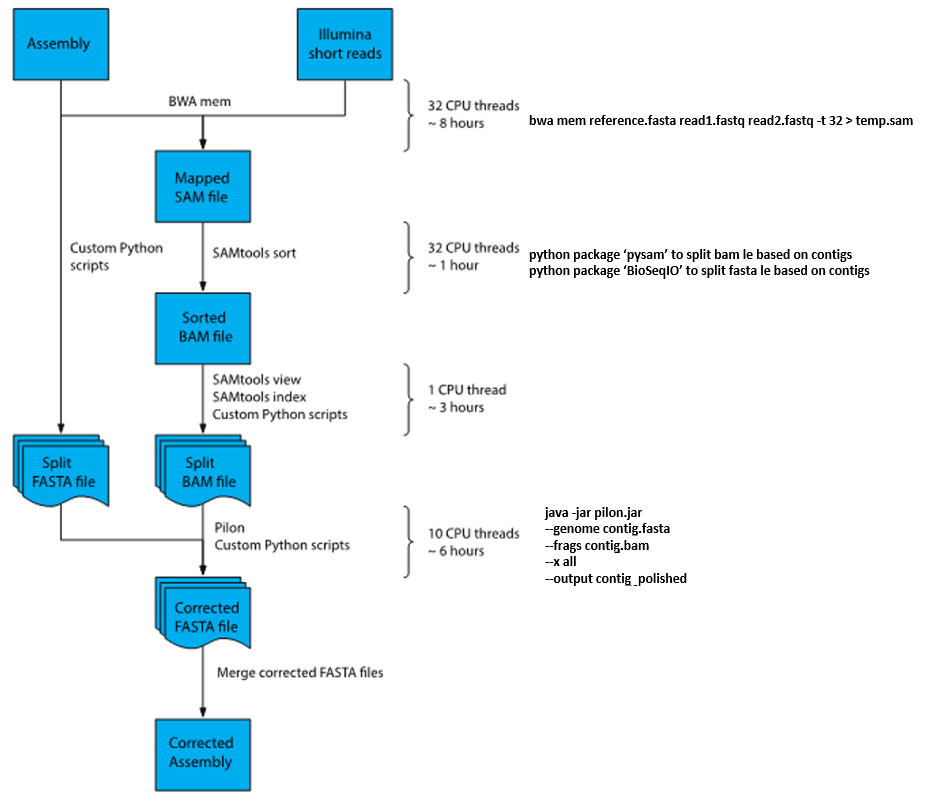

Supplement: Supplementary file 1 [file ijms-24-02904-s001.zip › Supplementary Figure S6.PNG]
